# Assessing population changes of historically overexploited black corals (Order: Antipatharia) in Cozumel, Mexico

**DOI:** 10.7717/peerj.5129

**Published:** 2018-07-04

**Authors:** Erika Gress, Dominic A. Andradi-Brown

**Affiliations:** 1 Nekton Foundation, Begbroke Science Park, Begbroke, Oxfordshire, United Kingdom; 2 Conservation Leadership Programme, Cambridge, United Kingdom; 3 Department of Zoology, University of Oxford, Oxford, United Kingdom; 4 Ocean Conservation, World Wildlife Fund - US, Washington, D.C., United States of America

**Keywords:** Mesophotic coral ecosystem, Antipatharian, Precious coral, Black coral, Harvest management, *Antipathes caribbeana*, *Plumapathes pennacea*, Caribbean, Cozumel Mexico, Jewellery industry

## Abstract

Black corals (Antipatharians) are crucial structural and ecological components of many mesophotic coral ecosystems (MCEs; reefs 30–150 m depth). In Mexico, black corals are harvested for the jewellery industry, which has historically led to population depletion. Harvesting began in the early 1960s and was concentrated around Cozumel Island until 1995. Since then, harvesting permits have been granted only for the mainland coast. Here we compare Cozumel populations between 1998 and 2016 for the two black coral species targeted by the jewellery industry. We found that densities of *Plumapathes pennacea* in 2016 were substantially lower than in 1998. However, the 2016 *P. pennacea* population has shifted to be dominated by larger colonies, suggesting disproportionate juvenile mortality or recruitment failure. Low numbers of *Antipathes caribbeana* were recorded, and no change in population density or colony size was detected between 1998 and 2016. Despite harvesting occurring for almost 70 years in the Mexican Caribbean, no information on reproduction, recruitment and other dynamics of the targeted species is available. We advocate for harvesting permits to be based on scientific evidence, and for implementation of future restrictions to prevent total depletion of black corals in the area.

## Introduction

Mesophotic coral ecosystems (MCEs; reefs 30–150 m depth) have attracted more research attention in recent years, and are likely to play an important role for the overall reef resilience. Still, they remain under-studied because of technical, logistical and financial challenges associated with surveying at their depth range (Hinderstein et al., 2010; Loya et al., 2016). Hard corals (Scleractinian) inhabit MCEs, particularly at upper-mesophotic (30–60 m) depths; although in many cases they are not the dominant benthic taxa (Sinniger et al., 2016). Substantial MCE habitat and structural complexity is provided by other ecosystem engineers such as calcareous macroalgae, octocorals, sponges, and black corals (Antipatharians) (Kahng et al., 2010; Bavestrello et al., 2014; Baker et al., 2016; Morais & Maia, 2016). MCEs communities can receive protection from some threats affecting shallow reefs, such as cyclones and sedimentation (Bridge et al., 2013). However, MCEs are known to face threats in their own right (Andradi-Brown et al., 2016), including overexploitation of economically important organisms such as fishes (Wood, Suliansa & Mustapa, 2006; Reed, Koenig & Shepard, 2007) and precious corals (including black corals) (Wells, 1981; Wagner, Luck & Toonen, 2012; Bruckner, 2016).

Black corals (Phylum Cnidaria, Class: Anthozoa, Order: Antipatharia) occur in all oceans from shallow (4 m) to abyssal depths (8,900 m), although are thought to be more common in tropical and subtropical regions at >50 m depths (Tsounis et al., 2010; Wagner, Luck & Toonen, 2012; Brugler, Dennis & France, 2013). Densities of black coral colonies are known to be influenced by currents speed, sedimentation and water quality, and the availability of hard substrate (Wagner, Luck & Toonen, 2012). About 250 black coral species have been described (Brugler, Dennis & France, 2013). Antipatharians are ahermatypic corals that depend on zooplankton as their major food source (Tsounis et al., 2010; Wagner, Luck & Toonen, 2012). They have annual vertical growth ranging between 1.2 cm, for a fan-shaped species, up to 159 cm for a wire-like species (Wagner, Luck & Toonen, 2012). Black corals life spans are also highly variable, ranging from 12 years to 4,250 years (Wagner, Luck & Toonen, 2012). Annual reproductive cycles culminating in the warmer months of the year have been reported for different species (Grigg, 1976; Wagner, Luck & Toonen, 2012). Gonochorism (male and female organs in different individuals) has been documented for most of the antipatharians, and internal fertilization has never been observed (Wagner, Luck & Toonen, 2012; Brugler, Dennis & France, 2013). However, long-term studies on black coral reproduction are very sparse (Wagner, Luck & Toonen, 2012).

On some MCEs, black corals are crucial habitat-forming species to which fish and other invertebrates associate with because of their complex structure and their ability to grow on steep walls or form dense beds (Boland & Parrish, 2005; Wagner, Luck & Toonen, 2012; Bruckner, 2016). For example, Love et al. (2007) found >2,250 invertebrates living in a single black coral colony (*Antipathes dendrochristos*) in Southern California. In Hawaii, Pomacanthidae and Pomacentridae fishes can be resident within individual black coral colonies, and many other fishes use black coral branches for shelter (Boland & Parrish, 2005). Endangered Hawaiian monk seals (*Monachus schauinslandi)* have also been observed using black coral beds as foraging habitats (Parrish et al., 2002). These dense beds are sometimes described as underwater animal forests, where black corals provide most of the structural habitat at mesophotic depths (Morais & Maia, 2016).

Black corals have dark coloured skeletons (hence their common name) that are composed of chitin and scleroprotein (Brugler, Dennis & France, 2013), which has been used for the jewellery industry since ancient times ( Grigg, 1993; Bruckner, 2016). Overexploitation of black corals for use in the jewellery industry has led to harvesting regulations in some locations (Grigg, 2001; Boland & Parrish, 2005; Bruckner, 2016; Todinanahary, Terrana & Lavitra, 2016). However, scientifically based guidelines for size and/or weight limits (maximum sustainable yields) are only known from Hawaii (Grigg, 1976; Grigg, 1984; Grigg, 2001; Grigg, 2004). There, the maximum sustainable yield has been determined using estimates of demographic rates such as recruitment, growth and mortality (Grigg, 1984). At a global scale, all black corals were included in the Convention on International Trade in Endangered Species of Wild Flora and Fauna (CITES) Appendix II in 1981 (CITES 2017). Therefore, countries (party to CITES) allowing exports of black corals are required to make an assessment to determine that harvesting ‘will not be detrimental to the survival of that species’ (CITES 2017). However, many countries have weak or no implementation of these regulations (Grigg, 1984; Bruckner, 2016).

In the Mexican Caribbean, harvesting of black corals began in the early 1960s (Kenyon, 1984), and has depleted black coral populations over wide geographical areas (Padilla & Lara, 2003; Padilla Souza, 2004). In 1994 three black coral species were added to the Mexican national protected species list (NOM-059) (Padilla & Lara, 2003): *Antipathes bichitoena*, *A. grandis* and *A. ules*. Species authorities were not included in the listing and taxonomy has changed, but it is believed *A. bichitoena* refers to *A. dichotoma* (Pallas, 1766), *A. grandis* (Verrill, 1928), and *A. ules* refers to *Myriopathes ulex* (Ellis & Solander, 1786) (Padilla Souza, 2004). No in-water studies of black corals had been conducted in Mexico prior to this listing. These three species were added in the original list by international recommendation because of the concern harvesting could lead to population depletion, which had been reported elsewhere (Wells, 1981; Wagner, Luck & Toonen, 2012; Bruckner, 2016). The inclusion of these species was a mistake, as they have not been recorded from the Mexican Caribbean or Mexican Pacific coasts, and based on their known distributions are unlikely to be found in Mexican waters (WoRMS, 2004; WoRMS, 2008a; WoRMS, 2008b).

The first Mexican black coral surveys were conducted in 1998–1999 in the Mexican Caribbean along the mainland coast, in Cozumel Island and in Chinchorro Atoll, to a maximum depth of 80 m (Padilla Souza, 2000). Eight black coral species were recorded in the area and taxonomy was confirmed by experts (Padilla Souza, 2000): *A. lenta* (Pourtalés, 1880), *A. atlantica* (Gray, 1857), *Cupressopathes gracilis* (Thomson & Simpson, 1905), *Stichopathes lutkeni* (Brook, 1889), *Tanacetipathes hirta* (Gray, 1857), *T. tanacetum* (Pourtalès, 1880); and the two harvested species were identified as *A. caribbeana* (Opresko, 1996) and *Plumapathes pennacea* (Pallas, 1766) (Padilla, 2001; Padilla & Lara, 2003). Despite realisation of the mistake on the Mexican protected species list, the original 1994 listing has not been updated, resulting in no national level protection for any recorded Mexican black coral species. However, all commercial fisheries activities, including black coral harvesting, require official permits.

The first record of official black coral harvesting permits granted in Mexico dates to 1976. Independently of the protection status of an area, harvesting permits can be obtained from the Mexican fisheries department. More strict protocols should be follow prior to issuing permits for species listed in the Mexican protected species list. Black coral harvesting permits do not specify species, the general term ‘*coral negro’* (black coral) is used and they are usually valid for two years. They only specify the grantees, the extraction area, and the weight limit (varying between 50–150 kg/month) (Padilla, 2001). Permits restrictions and extraction areas have been mainly based on fisheries requests (Padilla, 2001). Moreover, there is no evidence of enforcement to ensure commercial harvesting is correctly complying with permit restrictions. From 1976–1995 permits were only granted to extract black corals in Cozumel Island. Since 1995 until the present (2018) permits have been granted for different locations along the Mexican mainland coast, with no additional permits for Cozumel.

**Figure 1:**
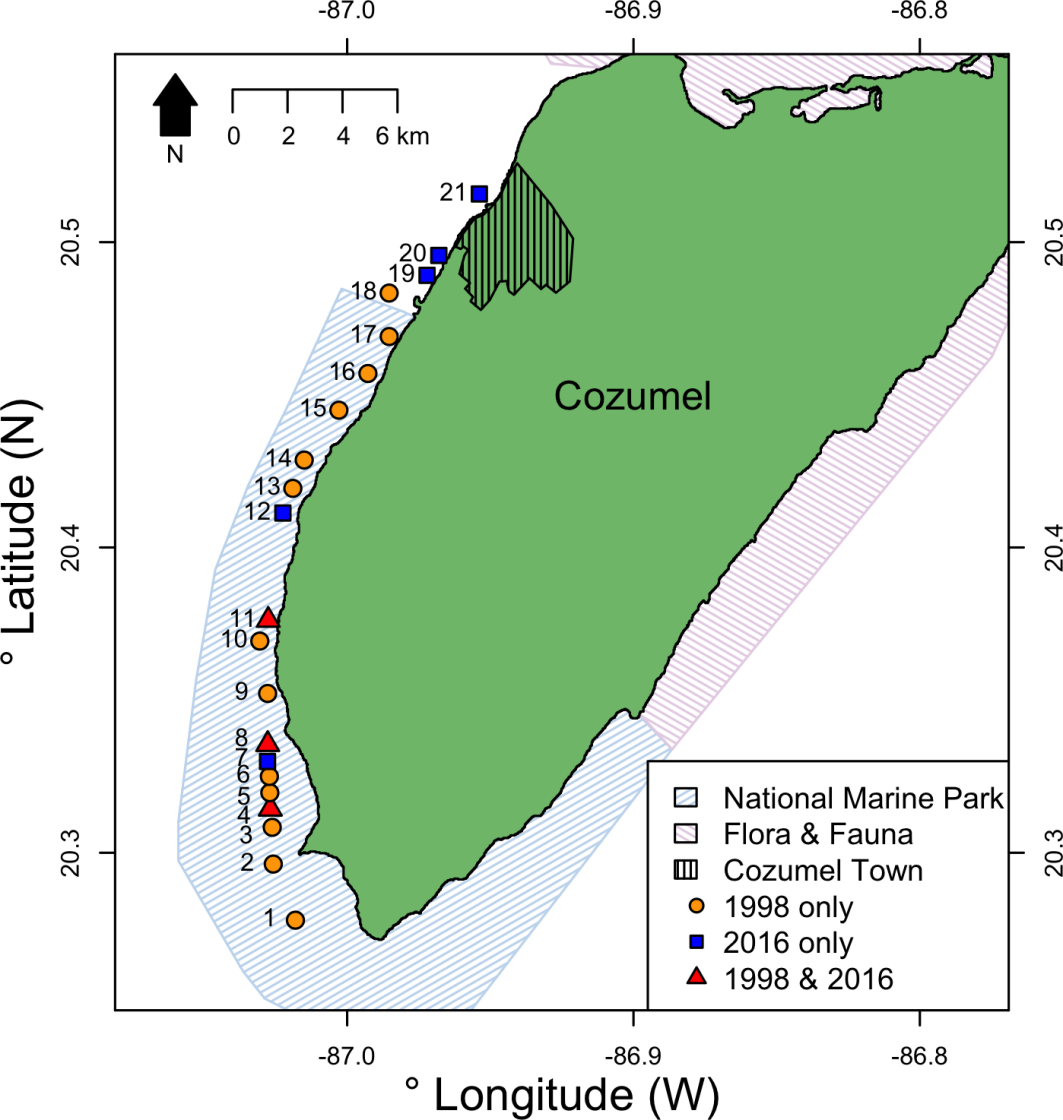
Location of survey sites relative to Cozumel and the National Marine Park and Flora & Fauna protected areas on Cozumel. Years when surveys were conducted are indicted by shape and color. Sites (GPS locations in Data S1) were: 1, Maracaibo; 2, Punta Sur; 3, Colombia 1; 4, Colombia 2; 5, Estacion de Monitoreo en Cozumel; 6, Colombia North; 7, Herradura; 8, Palancar Jardines; 9, El Cedral; 10, Paso el Cedral; 11, Santa Rosa; 12, Punta Tunich; 13, Yucab; 14, Tormentos; 15, Chancanab; 16, Las Palmas; 17, Paraiso; 18, Caleta; 19, Villa Blanca; 20, Transito Transbordador; 21, Purgatorio.

Cozumel is a small Caribbean island 17 km off the north-eastern Yucatan peninsula (Fig. 1). On the western side of the island, the shelf edge lies at 20 m, the insular slope drops at an angle of 70°–80°, and this steep slope ends at around 400 m depth ( Muckelbauer, 1990). The slope is a continuous wall influenced by strong currents flowing from south to north (Chávez, Candela & Ochoa, 2003), where mesophotic reefs are well developed (Gress et al., 2017). The main benthic communities at the upper-mesophotic (30–60 m depth) are macroalgae, gorgonians, sponges and black corals (Gress et al., 2017). The southwest of Cozumel (Fig. 1) was decreed a National Marine Park (MPA) in 1996 (Diario Official 1998). The MPA is zoned and allows fishing on specific areas with non-intensive use (i.e., areas usually beyond the 50 m isobath where less tourism activities take place).

Historically, Cozumel was famed for having extensive and highly dense black coral populations on MCE reef walls. The island remains as the major production and sales centre for the Mexican black coral jewellery and handcraft industry (Kenyon, 1984; Padilla, 2001); and both targeted species have the same market value (Padilla, 2001). There is no baseline data of pre-harvesting black coral population densities from Cozumel. However, there is relevant historical data that reports the decline (La Torre Alegria De, 1979; Kenyon, 1984; Humann & Deloach, 2001; Padilla, 2001). Harvest rates from Cozumel in the mid-1970s were between 70–121 kg gross black coral product per year (La Torre Alegria De, 1979). By the late 1980s and early 1990s rates had risen to between 1,000–1,500 kg per year. Such harvesting intensity was followed by a population decline that collectors reported (Padilla Souza, 2004; Padilla & Lara, 2003). Mexican authorities suspended permission for black coral extraction in Cozumel in 1995 citing collector safety, as commercial sized colonies had reportedly been depleted to >75 m depth (La Torre Alegria De, 1979; Padilla Souza, 2004). This resulted in collectors adopting increasingly deeper high-risk bounce diving, whereas in the past they could harvest colonies from as shallow as 20 m (Padilla Souza, 2004). Since the 1995 no new permits have been issued for Cozumel. However, because of the rapid overexploitation of black coral on mainland, harvesters have expressed interest on obtaining permits to harvest in Cozumel again (E Gress, 2016).

Despite the reported decline in Cozumel black coral populations, no assessments were conducted until 1998 when surveys recorded black coral densities and colony height, width and stem diameter at 15 sites on the west coast of Cozumel (Padilla, 2001; Padilla & Lara, 2003; Padilla Souza, 2004). On MCEs at the southwest of Cozumel, black corals do not form dense beds separated by gaps (Padilla Souza, 2000; Gress et al., 2017), although black coral population densities were reported to be higher on the southwest of the island (Padilla Souza, 2000). It is not clear if this relatively uniform distribution represents the natural pre-harvesting condition, as in the northern Mexican Caribbean similar distribution has also been observed (Padilla Souza, 2000; Padilla Souza, 2004). This contrasts with southern Mexican mainland coastal areas and the Chinchorro Atoll where dense aggregations of black coral colonies were recorded (Padilla Souza, 2004; Padilla & Lara, 2003).

Here we report a new black coral population assessment conducted around Cozumel during 2016. We compare changes in the population densities and size distribution of the two historically harvested species of black coral, *A. caribbeana* and *P. pennacea*, on MCEs between 1998 and 2016. In the absence of black coral harvesting permits being issued for Cozumel since 1995, we hypothesise the populations will be recovering. Given the population depleted of Cozumel black coral populations in 1998, we would expect population recovery to be apparent though increases in black coral colony density concurrent with increases in larger sized colonies. We evaluate current population trajectories to inform local and national managers.

## Methods

### Black coral surveys

Surveys were conducted at eight sites on the west coast of Cozumel, Mexico during August–September 2016. Five sites were within the MPA, and three were in an area with no protection adjacent to the main town. MPA sites were Santa Rosa, Colombia, Punta Tunich, Palancar Jardines and Herradura, and non-MPA sites were Transito Transbordador, Purgatorio and Villa Blanca (Fig. 1).

Black coral surveys were conducted using a diver-operated stereo-video system (stereo-DOV), consisting of two cameras separated by 0.8 m and with approximately 3°convergence angle filming forward along the reef. A stereo-DOV system records two synchronised images of the reef, allowing accurate length measurements of reef benthic organisms (Turner et al., 2015; Bennett et al., 2016). The stereo-DOV used two GoPro Hero 4 Black cameras and a spool system with biodegradable line for measuring out each transect (see (Gress et al., 2017) for details). Transects were 30 m in length, conducted parallel to the coast following the current direction, and each separated by 10 m intervals. At each site, four transects were conducted at 55 m depth, giving 32 transects in total across all eight sites. Transects were filmed during daylight hours using natural ambient light. When filming transects, the stereo-DOV operator swam with the cameras recording forward along the reef at the 55 m depth contour while carefully looking for colonies. Upon encountering a black coral, the operator slowed and angled the cameras to ensure the coral was captured clearly on both cameras. Permits for surveys were issued by the Comisión Nacional de Áreas Naturales Protegidas (CONANP) Cozumel, Permit Number: FOO.9.DPNAC/305-16.

We also obtained the raw Cozumel data from Padilla Souza (2004), which contains densities and colony sizes for *A. caribbeana* and *P. pennacea* from 15 sites from the west coast of Cozumel (Fig. 1). Their studies were conducted between June 1998 and September 1999 (collectively referred to as 1998 in this study). These surveys were conducted by open-circuit divers at each site and spanned from 18–80 m depth, though precise depths differed for each site. Divers descended to their maximum survey depth, and then slowly ascended recording the height, width and depth of each black coral colony encountered by using a measuring tape, trying to keep survey effort roughly equal across the depth gradient (Padilla Souza, 2000; Padilla Souza, 2004). The total area of reef surveyed at each site along the depth gradient was estimated. No transects were defined, and there is no record of the exact area surveyed at each depth.

### Analysis

Stereo-DOV footage was analysed using EventMeasure (v4.42, SeaGIS, Melbourne, Australia). All *A. caribbeana* and *P. pennacea* colonies within a 4 m transect width (constrained using EventMeasure) were identified, giving a total density for each species per 120 m^2^ transect. Species identification was done following Opresko & Sánchez (2005). The maximum height and maximum width of each colony was measured using EventMeasure built in length measurement tools.

As sites surveyed in 1998 and 2016 were not all the same, we used an analysis of covariance (ANCOVA) to detect changes in black coral density between years controlling for potential differences based on site location along the west coast of Cozumel. Initially we identified three covariates reported to influence black coral density (Wagner, Luck & Toonen, 2012): (i) current strength from the Bio-ORACLE database (Assis et al., 2018), (ii) coastal development, and (iii) marine based pollution from the Reefs at Risk Revisited report (Bryant et al., 1998). Current strength represented mean current strength at mean depth for each of the sites. Coastal development and marine based pollution was allocated scores from 1–4 with larger values indicating greater threats. Coastal development represents the size and density of hotels, cities, ports, airports, and coastal population adjacent to reef sites. Marine-based pollution represents the size and volume of commercial shipping ports and cruise ship ports, the intensity of shipping traffic, and the location of oil infrastructure adjacent to the reefs. In addition, we included the latitude of sites as an additional factor to account for any additional variation along the western coast of Cozumel. Black coral density data was square root transformed prior to analysis to ensure model assumptions were met. To assess the statistical power of this study for detecting differences in black coral density between years, we also conducted a power analysis using the ‘pwr.t2n.test’ function from the pwr package (Champely, 2018). This was based on the differing number of survey sites in each year, and our recorded black coral densities and associated standard deviations for each year.

Changes in colony size (maximum height and maximum width) were tested using kernel density estimates (KDEs) and permutation tests, following Langlois et al. (2010). This method allows differences between two length distributions to be tested, and provides information of where in the length distributions any significant differences are located. KDEs were fitted separately to the two groups with the Sheather-Jones selection procedure (Sheather & Jones, 1991) using the ‘KernSmooth’ package (Wand, 2013), and plotted. A permutation test then randomly allocated the data into two groups, and the mean and standard error of these randomly allocated distributions can be plotted. The permutation test was run for 9999 permutations, and used the function ‘sm.density.compare’ in the package ‘sm’ (Bowman & Azzalini, 2014), in R (R Core Team, 2013). As 2016 data was limited to transects at 55 m depth, while the 1998 data incorporated colonies surveyed from 18–80 m depth, we tested for changes in colony size with depth within the 1998 data using linear models. Linear model residual plots were inspected to ensure model assumptions were not violated. Permutation tests comparing changes in colony size between years were run comparing 2016 surveys with all 1998 data, and just colonies recorded between 50–60 m depth in 1998. All data is contained in Data S1, S2 and R code for analysis in Code S1.

## Results

### Changes in black coral density

**Figure 2:**
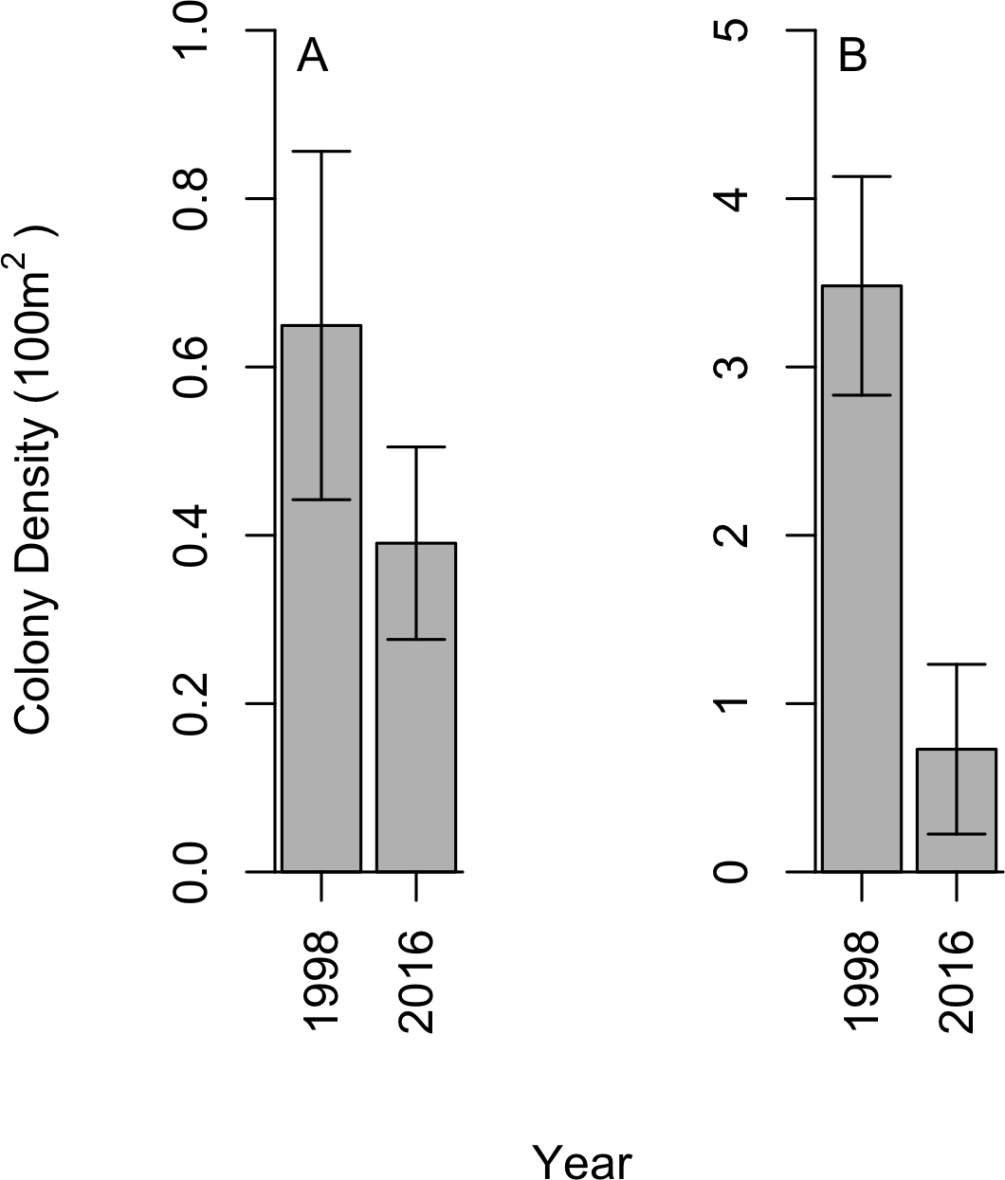
Change in black coral density between 1998 and 2016 for (A) *A. caribbeana*, and (B) *P. pennacea*. Error bars show 1 standard error above and below the mean.

We did not find black corals to be aggregated on dense beds along the Cozumel steep wall; instead, a uniform distribution was observed within sites, which aligns with the 1998 studies. In 2016, a total of 28 *P. pennacea* and 15 *A. caribbeana* colonies across all 32 transects were recorded. *P. pennacea* was more abundant than *A. caribbeana* in 2016, with mean densities of 0.73 ± 0.50 and 0.39 ± 0.11 per 100 m^2^ respectively (mean ± SE; Fig. 2). Although we found more variation in density between sites for *P. pennacea*, with standard deviation of 1.43 compared to 0.32 for *A. caribbeana*. Mean black coral colony density was lower for both *A. caribbeana* and *P. pennacea* in 2016 than 1998 (Fig. 2). Results showed a significant decline for *P. pennacea* although it was not significant for *A. caribbeana* (Table 1). We conducted a power analysis of our ability to detect a change in *A. caribbeana* density between years, finding low statistical power at 0.15 (Type II error rate: 85 %). Latitude significantly affected *A. caribbeana* density, with grater densities in the south of Cozumel. No effect of latitude was found on *P. pennacea* density (Table 1). The removal of interaction terms from the *P. pennacea* model reduced model AIC, therefore these interactions were retained despite not having significant p values (Table 1). No effects of marine based pollution were found on either species, and this variable was removed from the final models. We also compared 2016 black coral density between our five sites inside the Cozumel MPA and the three sites outside. *A. caribbeana* density was greater inside the MPA than outside (Mann–Whitney *U* = 14.5, *p* = 0.044), with 0.58 ± 0.10 colonies per 100 m^2^ inside the MPA compared to 0.07 ± 0.07 colonies per 100 m^2^ outside the MPA. There was no difference in *P. pennacea* density between sites inside the MPA and those outside (Mann–Whitney *U* = 9, *p* = 0.73).

### Changes in black coral colony size

**Figure 3:**
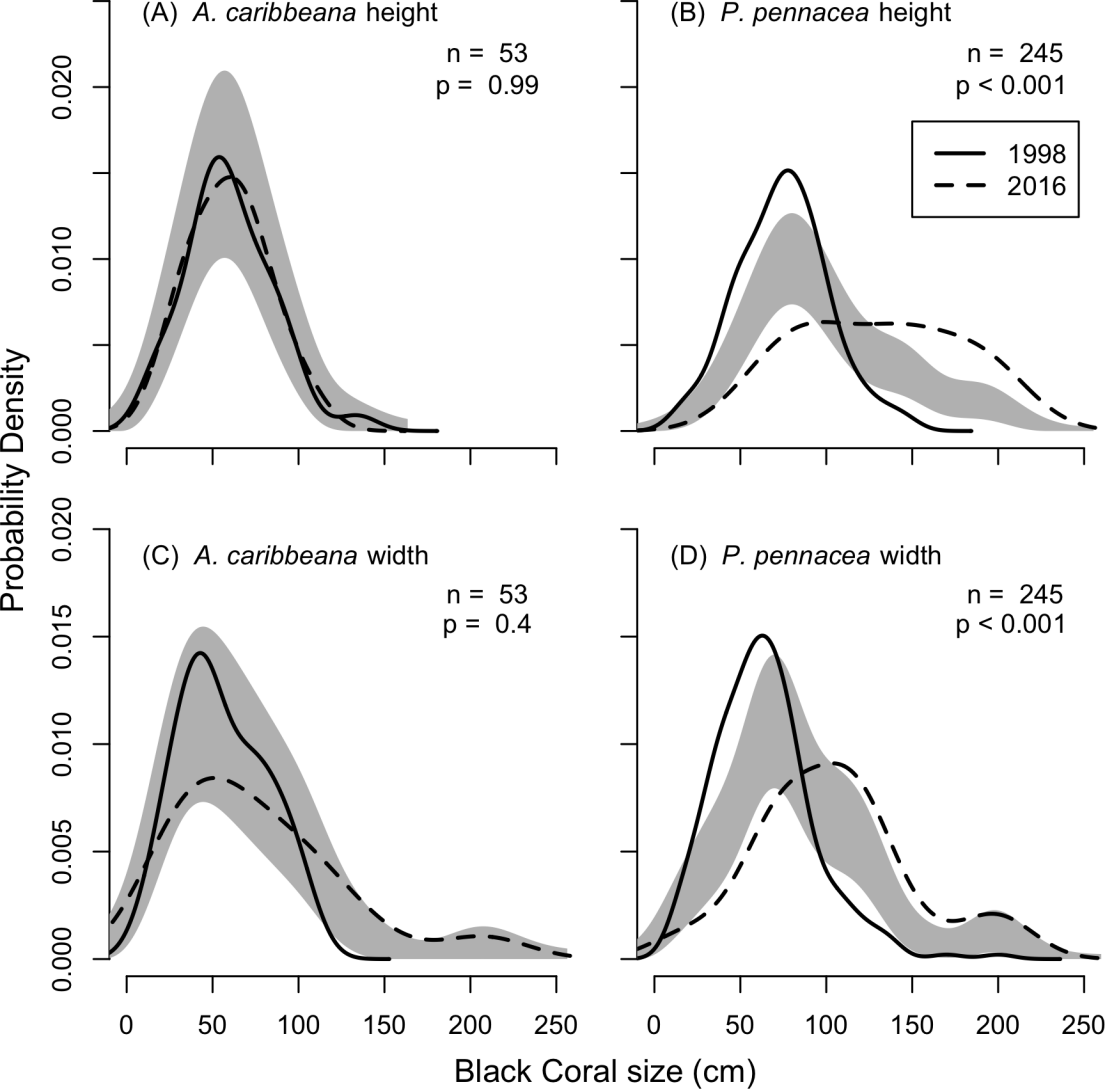
Change in black coral colony size between 1998 and 2016, for (A) *A. caribbeana* colony height, (B) *P. pennacea* colony height, (C) *A. caribbeana* colony width, and (D) *P. pennacea* colony width. Kernel density estimates were used, followed by a permutation test to identify differences between years. The grey shaded area indicates one standard error either side of the null model of no difference in colony size distribution based on year. Locations where the lines representing 1998 and 2016 are outside the grey zone indicate significant differences in the proportion of colonies of that size. *n*, number of colonies.

**Figure 4:**
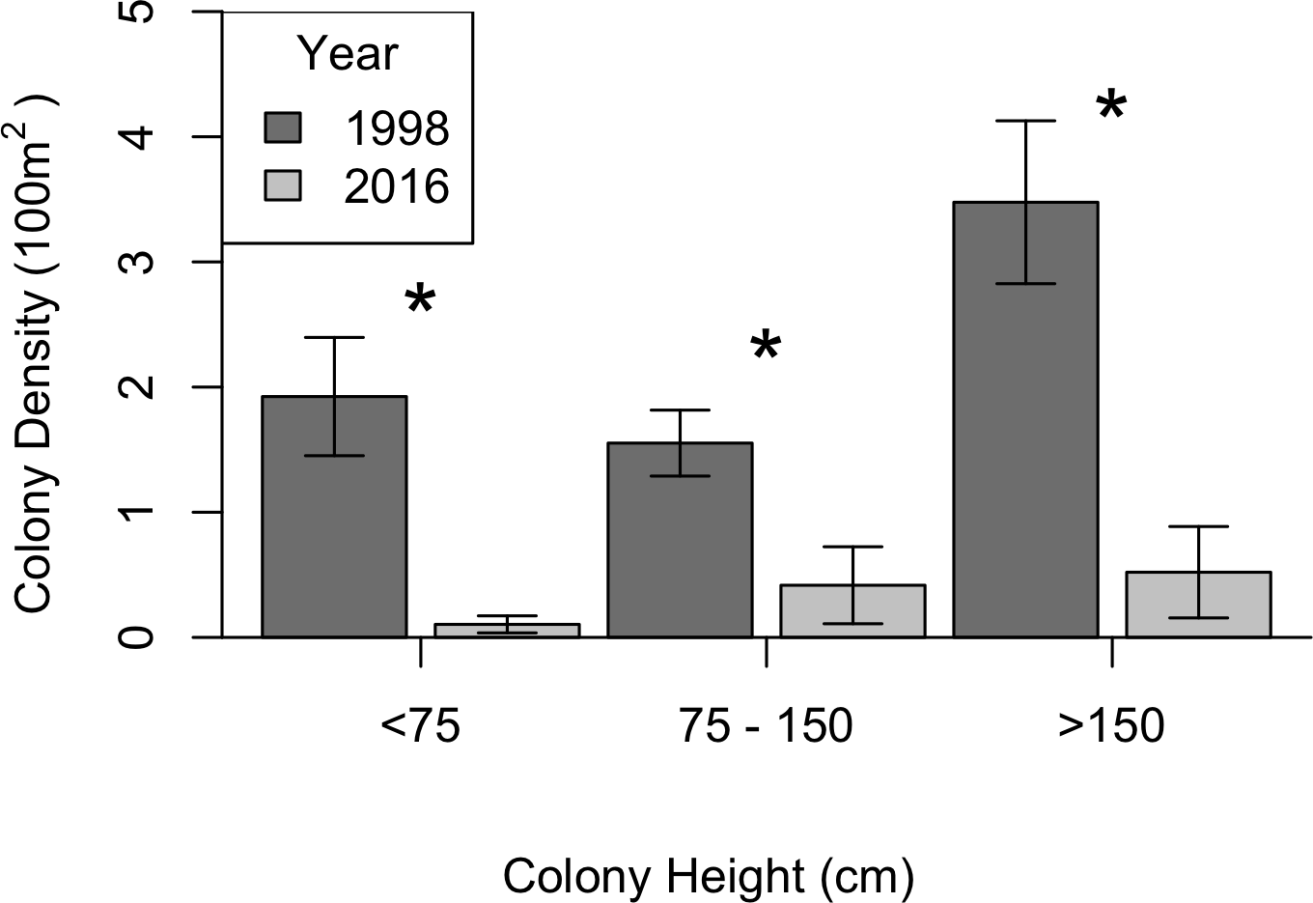
Change in black coral density between 1998 and 2016 for *P. pennacea* grouped by colony height. Stars indicate significant differences at *p* < 0.05 using Mann–Whitney *U* tests. Error bars show one standard error above and below the mean.

KDEs indicated that there was no change in *A. caribbeana* size between the two surveys, while *P. pennacea* colonies were larger in 2016 than 1998 (Fig. 3). *A. caribbeana* colony height was surprisingly consistent between years, with median colony heights of 59 cm in both 1998 and 2016 (Fig. 3A). There was a change in *A. caribbeana* median colony width, from 50 cm in 1998 to 66 cm in 2016, although this was not significant (Fig. 3C). In contrast, *P. pennacea* colonies were both taller (median: 75 cm in 1998, 134 cm in 2016; Fig. 3B) and wider (median: 61 cm in 1998, 105 cm in 2016; Fig. 3D) in 2016. To ensure *P. pennacea* colony size differences were not caused by losing small individuals from the population, we separated density data by height class, finding colonies of all three-height classes (<75 cm, 75–150 cm, >150 cm) declined in density between 1998 and 2016 (Fig. 4).

**Table 1:** ANCOVA results for changes in black coral density between 1998 and 2016.

Family/term	SS	DF	*F*	*P*
*A. caribbeana*				
Year	0.10	1	0.66	0.425
Current	0.45	1	3.04	0.097
Latitude	0.81	1	5.48	0.030
Year:Current	0.28	1	1.93	0.181
Residuals	2.80	19		
*P. pennacea*				
Year	8.76	1	21.70	<0.001
Current	0.92	1	2.28	0.150
Coastal development	0.05	1	0.11	0.742
Latitude	0.94	1	2.33	0.146
Year:Current	0.57	1	1.40	0.253
Year:Coastal development	0.26	1	0.64	0.437
Year:Latitude	1.68	1	4.17	0.058
Residuals	6.46	16		

As surveys in 2016 were conducted at 55 m, whereas surveys conducted in 1998 spanned 18–80 m depth we tested for effects of depth on colony size in the 1998 data. No depth changes in colony height (*F*_1,36_ = 1.7, *p* = 0.199) or width (*F*_1,36_ = 3.1, *p* = 0.089) were detected for *A. caribbeana*. However, *P. pennacea* colonies were both taller (*F*_1,215_ = 14.8, *p* < 0.001) and wider (*F*_1,215_ = 8.4, *p* = 0.004) at shallower depths (Fig. S1). Though there was high variability in colony size across this depth gradient, with low *R*^2^ values of 0.06 and 0.03 for height and width respectively (Fig. S1). To ensure differences identified in colony size between 1998 and 2016 were not caused by comparisons of colonies from different depths, we reran the size comparison analysis using only colonies recorded from 50–60 m depth in 1998 (Fig. S3). This restricted 50–60 m depth range analysis produced results highly consistent to those using the full 1998 dataset (Fig. S2; Fig. 3).

## Discussion

### Differences in black coral density

Black coral commercial harvesting has not been allowed anywhere in Cozumel since 1995; and the MPA was established one year later (1996). Therefore, we expected the 2016 surveys to identify stable or increasing black coral populations independently of the protection scheme. However, significant declines in *P. pennacea* density and trends towards lower density for *A. caribbeana* were observed for all size classes between 1998 and 2016. Our 2016 results imply that the current MPA and fisheries regulation implementation is insufficient to enable black coral population recovery.

Only the total area of reef surveyed at each site was recorded in 1998, with no record of the area surveyed at each depth. Divers tried to keep the survey effort roughly equal across the depth gradient, recording the depth of each individual colony identified. We plotted the number of colonies recorded at each depth for both species from the 1998 surveys, finding the greatest frequency of *P. pennacea* colonies between 50–60 m (Fig. S3). As surveys from 2016 were only conducted at 55 m depth, this could raise the concern that differences in density between years could be driven by natural variation in black coral colony density with depth. Although this cannot be ruled out definitively, as the highest frequency of *P. pennacea* was found in the 50–60 m depth band in 1998, we believe it is highly unlikely. In addition, Padilla & Lara (2003) states that the greatest black coral abundance for all species was observed at approximately 60 m. As there was roughly equal sampling effort across the depth gradient, and the greatest *P. pennacea* frequency was at 50–60 m, it is likely that the site densities from 1998 are underestimates of *P. pennacea* density at 50–60 m. Therefore, if 1998 density data was available broken down by depth, we would expect more severe declines in *P. pennacea* density than the ones detected.

Interpreting the possible influence of changing colony densities with depth on *A. caribbeana* is harder because of the low number of colonies recorded in 2016. In 1998 *A. caribbeana* greatest colony frequency was recorded at 60–70 m (Fig. S3). However, the 50–60 m depth range contained the second greatest frequency, and few colonies were encountered >70 m or <40 m (Fig. S3). In a similar way to *P. pennacea*, this also implies that 1998 *A. caribbeana* density in the 50–60 m range may have been higher than the overall site estimates. If this were the case it suggests *A. caribbeana* densities may have declined as well through time. However, this interpretation requires caution, as we only found 15 *A. caribbeana* colonies in 2016, and had low statistical power to identify differences in density between 1998 and 2016. Low densities of *A. caribbeana* around Cozumel were previously identified in the 1998 population assessment (Padilla Souza, 2004). *A. caribbeana* low density has been recorded across the Mexican Caribbean with the exception of Chinchorro Atoll (Padilla & Lara, 2003). Similarly, a study further south on the Mesoamerican Reef, in Honduras, recorded *P. pennacea* but found no colonies of *A. caribbeana* (Guzman, 1998).

Even with only some of the sites surveyed in 2016 identical to those in 1998, we believe that our results still provide valuable information on the status and trends in black corals around Cozumel. In 2016, we surveyed two more sites (compared to 1998) outside the MPA. All sites surveyed in 2016 were either surveyed in 1998 or are adjacent to sites that were surveyed in 1998 (Fig. 1). Black coral colonies in Cozumel are not aggregated in dense beds, but are instead evenly distributed along the steep MCE wall within sites, a feature also recorded in 1998 by (Padilla Souza, 2000). Therefore, there is low variation in colony density between different areas at the same depth within a site. We controlled for differences in black coral density based on site location by incorporating current strength, coastal development, marine based pollution, and latitude into our models (Table 1); factors which are known to influence black coral density (Wagner, Luck & Toonen, 2012). Therefore, we consider the comparison of population density between both years valid, since it was done estimating colonies per m^2^ in the same areas and we controlled for variables affecting black coral density.

### Differences in black colonies size

Our size distribution analysis implies that colonies of *P. pennacea* were both taller and wider on average in 2016 than in 1998, but no changes were detected for *A. caribbeana* (Fig. 3). At first glance this may suggest some *P. pennacea* colonies are recovering from the historical harvesting pressure and maturing. In the Mediterranean, other precious corals (e.g., *Corallium rubrum*) have exhibited populations (>50 m depth) of sparsely distributed large colonies, and no small or young colonies (Cau et al., 2016). This process known as ‘self-thinning’ is believed to occur due to intra-specific competition for space and is expected in populations at the maximum saturated density (Cau et al., 2016). Even though there is no pre-harvesting population density data available for Cozumel, there is historical information that describes a black coral population in decline. Three years before the first surveys were conducted in Cozumel in 1998, fishers asked for permits to harvest on other areas because of depletion of black corals colonies that could be easily accessed (Padilla Souza, 2000; Padilla Souza, 2004). Therefore, densities reported by Padilla Souza (2000) are most probably not representing a maximum saturated density for the two targeted species. The 2016 results show a lower density of large *P. pennacea* colonies than in 1998. Therefore, self-thinning is unlikely driving the changes observed in colony size.

The *P. pennacea* density decline across all height classes (Fig. 4), combined with a shift to larger colonies could suggest that juvenile colonies have been disproportionately affected by historical harvesting activities. However, this is unlikely, as larger colonies have greater commercial value and Padilla Souza (2000) reported high abundance of juvenile black corals and colonies regenerating from standing bases of previously harvested colonies in Cozumel. Disentangling possible causes for a disproportionate loss of smaller colonies in the population between 1998 and 2016 is complex, but implies reduced black coral recruitment or juvenile survival. There have been few long-term studies of black coral populations conducted, and the processes affecting black coral recruitment and juvenile survival are poorly understood (Wagner, Luck & Toonen, 2012). In Hawaii, black coral recruitment has declined (Grigg, 2004), most likely caused by overharvesting mature colonies (Grigg, 2004; Tsounis et al., 2010), though competition with an invasive species might also be involved (Grigg, 2004; Kahng & Grigg, 2005). This implies that while harvesting typically targets the largest colonies (Padilla & Lara, 2003), the decline in large colony density in Cozumel could be reducing juvenile recruitment rates.

Understanding the potential causes of *P. pennacea* loss and/or current population structure in Cozumel requires further research on reproduction, settlement and recruitment success; as well as of ecological and environmental parameters. There is currently no information about the reproductive method of either *A. caribbeana or P. pennacea*. Gonochorism and no internal fertilization have been reported for most of the studied black corals (Wagner, Luck & Toonen, 2012). If the case for *A. caribbeana* and *P. pennacea*, the low population density could be having implications on fertilization rates as shown for the Caribbean octocoral *Plexaura kuna* (Coma & Lasker, 1997). Fertilization rates of this broadcast spawning gorgonian dropped drastically with increased distance between female and male colonies, with underwater current speed also playing an important role in this decline (Coma & Lasker, 1997). Therefore, it is possible that black corals are suffering from an Allee effect (Courchamp, Clutton-Brock & Grenfell, 1999), with the low density of black corals preventing fertilisation.

Other factors on Cozumel MCEs could play a part in *P. pennacea* size distribution shifts and density declines. Black corals need hard substrate for recruitment and to firmly attach onto for growth (Wagner, Luck & Toonen, 2012), and in the Caribbean are generally associated with steep outer reef slopes (Sanchez, Zea & Diaz, 1998). In Cozumel, surveys down to 33 m depth in the 1980s recorded mean macroalgal cover at 25% (Jordán Dahlgren, 1988), but surveys at 55 m in 2016 recorded mean macroalgal coverage at 44% (Gress et al., 2017). If macroalgal cover has increased, it could reduce substrate availability for *P. pennacea* recruitment. *P. pennacea* settlement has been studied in Jamaica, where areas adjacent to unstable sediment beds had lower settlement rates (Oakley, 1988). On Cozumel, shallow reef scleractinian cover has dropped from 44% to as low as 4% at some sites, caused by coastal development (Reyes-Bonilla, Millet-Encalada & Alvarez-Filip, 2014). It is unknown if reduced availability of substrate for recruitment could also be affecting MCEs and therefore black coral recruitment. *A. caribbeana* densities were also higher inside the MPA in 2016 than at sites outside the MPA (adjacent to the main coastal development where there has been the greatest shallow reef loss).

Illegal harvesting of black corals may also be a problem around Cozumel. Recent images and videos posted on social media show divers collecting black corals from locations identifiable as Cozumel reefs (E Gress, 2017). Though there is no data available on the frequently of occurrence, and illegal harvesting might contribute to the declines in *P. pennacea* density we report, we do not believe this is the primary driver of decline. Harvesting typically targets larger colonies (Padilla Souza, 2000), and we found disproportional declines in smaller colonies. Further research is required to understand factors causing the reduced densities of Cozumel black coral colonies.

### Management status

The Mexican government continues to issue commercial black coral harvesting permits stating the harvesting locations allowed. Since 1995, when harvesting permits ceased being issued for Cozumel, harvest locations on the mainland coast have changed regularly due to rapid black coral depletion (Padilla & Lara, 2003). A harvesting permit is currently issued until October 2018 for locations in the southern Mexican Caribbean. However, prior to our study, no black coral monitoring has been conducted in Mexico since 1998–1999 (Padilla Souza, 2000; Padilla Souza, 2004; Padilla & Lara, 2003). As all black corals are CITES Appendix II listed, the Mexican government is committed to ensure that black coral harvesting for export ‘will not be detrimental to the survival of that species’, and ‘export of specimens of any such species should be limited in order to maintain that species throughout its range at a level consistent with its role in the ecosystems’ (CITES 2017). Yet, harvest areas are currently designated based on diver and industry requests following harvest depletion, rather than harvest sustainability (Padilla & Lara, 2003). The CITES Trade database (http://www.trade.cites.org) contains Mexican black coral export records for the jewellery industry up to 2016, although the quantity of black coral items reported can be as low as 1 per year. These low number or reports might be because the major market for the jewellery in Cozumel is sales to tourists, who are unlikely to obtain CITES export permits, rather than large commercial exports for sale internationally. With no black coral population assessments conducted since 1998–1999, and no studies on recruitment rates or any other biological and ecological traits, it is unclear how the Mexican government is currently evaluating sustainability to continue issuing harvest permits and CITES export permits.

Moving forward, we acknowledge that effective implementation of regulations is indispensable for achieving positive effects on protected species or ecosystems. The inclusion (or correction) of the two targeted black corals in the Mexican protected species list could help promote advances for black corals protection in the country. Their inclusion in the Mexican protected species list should require more detailed information on the targeted species before harvesting permits could be issued, and make illegal harvesting of black corals a more serious criminal offence. In addition, adding these targeted black coral species to the Mexican threatened species list will also force a review of the legal status of the existing harvesting permits. As other well-managed black coral fisheries have struggled to maintain long-term sustainability (Tsounis et al., 2010; Bruckner, 2016), there is an urgent need to evaluate the biological and economic sustainability of the industry. Evidence from the few available reports show that unregulated and uninformed harvesting of black corals have quickly lead to overexploitation and population depletion in many areas in the Caribbean (Bruckner, 2016). Our results suggest that even following harvesting bans, Mexican black coral populations that have been heavily exploited are unlikely to recover. The Mexican government has recently announced a large MPA that includes most of the Mexican Caribbean. We strongly recommend that consideration is given to protect MCEs and their ecosystem engineers, such as black corals.

We also encourage urgent research to understand drivers of black coral population decline both within Cozumel and the Mexican Caribbean, but also the wider western Atlantic region. If recruitment failure is identified as the major driver of decline, it will be crucial to identify the exact mechanism. For example, a lack of fertilisation success could be addressed by direct black coral population restoration through transplantation (Montgomery, 2002). This restoration could target increasing black coral population densities to overcome any density-dependant recruitment limitation at sites that historically were sources of black coral recruits, to help black coral populations recover over larger areas. Alternatively, if reduced substrate for recruitment is limiting recruitment success, then management should focus on addressing the causes of this. For example, algal overgrowth of substrate could be tackled by management efforts to increase MCE herbivore populations, or sediment smothering of substrate could be addressed through improved coastal pollution management.

## Conclusion

We surveyed black coral populations around Cozumel finding severe declines in density between 1998 and 2016 for the historically most abundant species, *P. pennacea*. These declines affected corals of all size classes, though appeared to disproportionately affect smaller colonies. We highlight the urgent need to conduct studies to understand the causes of these trends, and also to assess the potential of biological and economical sustainability of black corals harvesting.

##  Supplemental Information

10.7717/peerj.5129/supp-1 Figure S1 Differences in *P. pennacea* colony (A) height and (B) width with depth from the 1998 surveys Solid red line shows linear model, while the dashed red lines show 95% prediction intervals. *P. pennacea* colonies were both taller (*F*_1,215_ = 14.8, *p* < 0.001) and wider (*F*_1,215_ = 8.4, *p* = 0.004) at shallower depths, though *R*^2^-values were 0.06 and 0.03 for height and width respectively.

10.7717/peerj.5129/supp-2 Figure S2 Change in black coral colony size between 1998 and 2016, using only colonies recorded between 50–60 m depth in 1998 (A) *A. caribbeana* colony height, (B) *P. pennacea* colony height, (C) *A. caribbeana* colony width, and (D) *P. pennacea* colony width. Kernel density estimates were used, followed by a permutation test to identify differences between years. The grey shaded area indicates one standard error either side of the null model of no difference in colony size distribution based on year. Locations where the lines representing 1998 and 2016 are outside the grey zone indicate significant differences in the proportion of colonies of that size. *n*, number of colonies.

10.7717/peerj.5129/supp-3 Figure S3 Frequency distribution showing the number of black coral colonies recorded in 1998 in 10 m intervals across the depth gradient for (A) *A. caribbeana*, and (B) *P. pennacea* Survey effort was approximately equal at different depths in 1998 (Padilla Souza, 2000; Padilla Souza, 2004).

10.7717/peerj.5129/supp-4 Code S1 Raw R code for the data analysis and figure generation for this study

10.7717/peerj.5129/supp-5 Data S1 Locations of surveyed sites and sites covariates from 1998 and 2016 Column headings are as follows: Long (longitude of surveyed sites in decimal degrees in WGS84 format), Lat (latitude of the surveyed sites in decimal degrees in WGS84 format), Year (the year of surveys), Name (full site name and survey date), Site (site name as in Data S2), Coastal.Dev (threat index from Reefs at Risk report), Marine.Threat (threat index from Reefs at Risk report), Current (mean current strength at mean depth for each of the sites).

10.7717/peerj.5129/supp-6 Data S2 Raw black coral data from 1998 and 2016 Column headings are as follows: Year (the year of survey; all 1998 and 1999 are labeled 1998), Survey.Year_Month (the year_month that the survey was conducted), Site (the Cozumel site name where surveys were conducted), Transect (the replicate transect number of the survey), Transect.Area. *m*^2^ (the survey area that the transect covered in *m*^2^), Height.cm (the black coral colony maximum height in cm), Width.cm (the black coral colony maximum width in cm), Diameter.cm (the black coral colony maximum stem diameter in cm), Depth (the depth the colony was recorded at in m), Genus (the colony genus name), Species (the colony species name).

## Additional Information and Declarations

### Field Study Permissions

The following information was supplied relating to field study approvals (i.e., approving body and any reference numbers): Field surveys were approved by Comision Nacional de Areas Naturales Protegidas, Cozumel (Permit Number: FOO.9.DPNAC/305-16).

### Data Availability

The following information was supplied regarding data availability: The raw data and code are available in the Supplemental File.
