# Supplementary figures and images for: Assessing population changes of historically overexploited black corals (Order: Antipatharia) in Cozumel, Mexico
Source: PeerJ. 2018 Jul 4;6:e5129. doi: 10.7717/peerj.5129 (PMC6035717; doi:10.7717/peerj.5129)

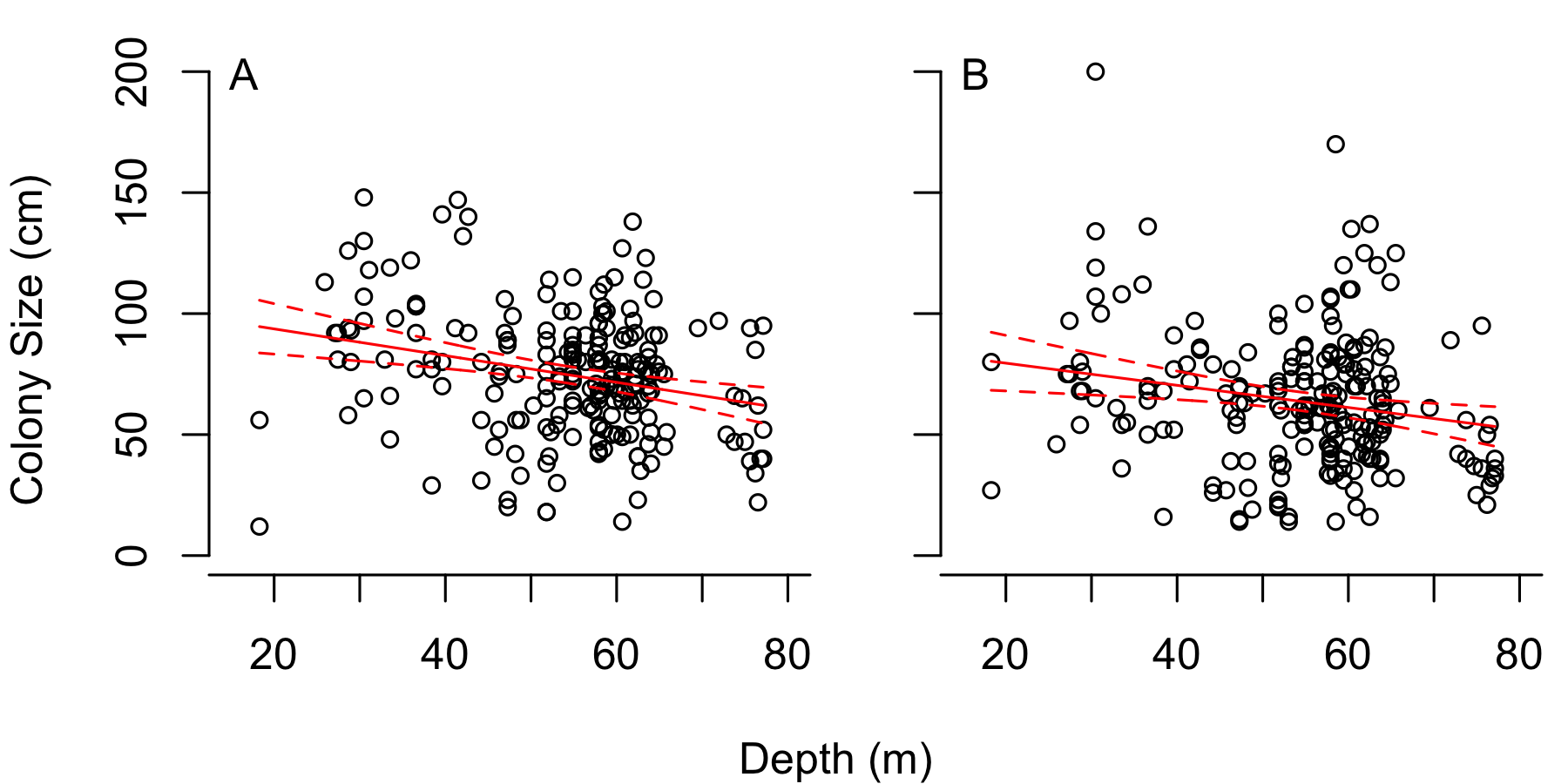

Supplement: Figure S1 — Solid red line shows linear model, while the dashed red lines show 95% prediction intervals. P. pennacea colonies were both taller (F1,215 = 14.8, p < 0.001) and wider (F1,215 = 8.4, p = 0.004) at shallower depths, though R2-values were 0.06 and 0.03 for height and width respectively. [file peerj-06-5129-s001.png]

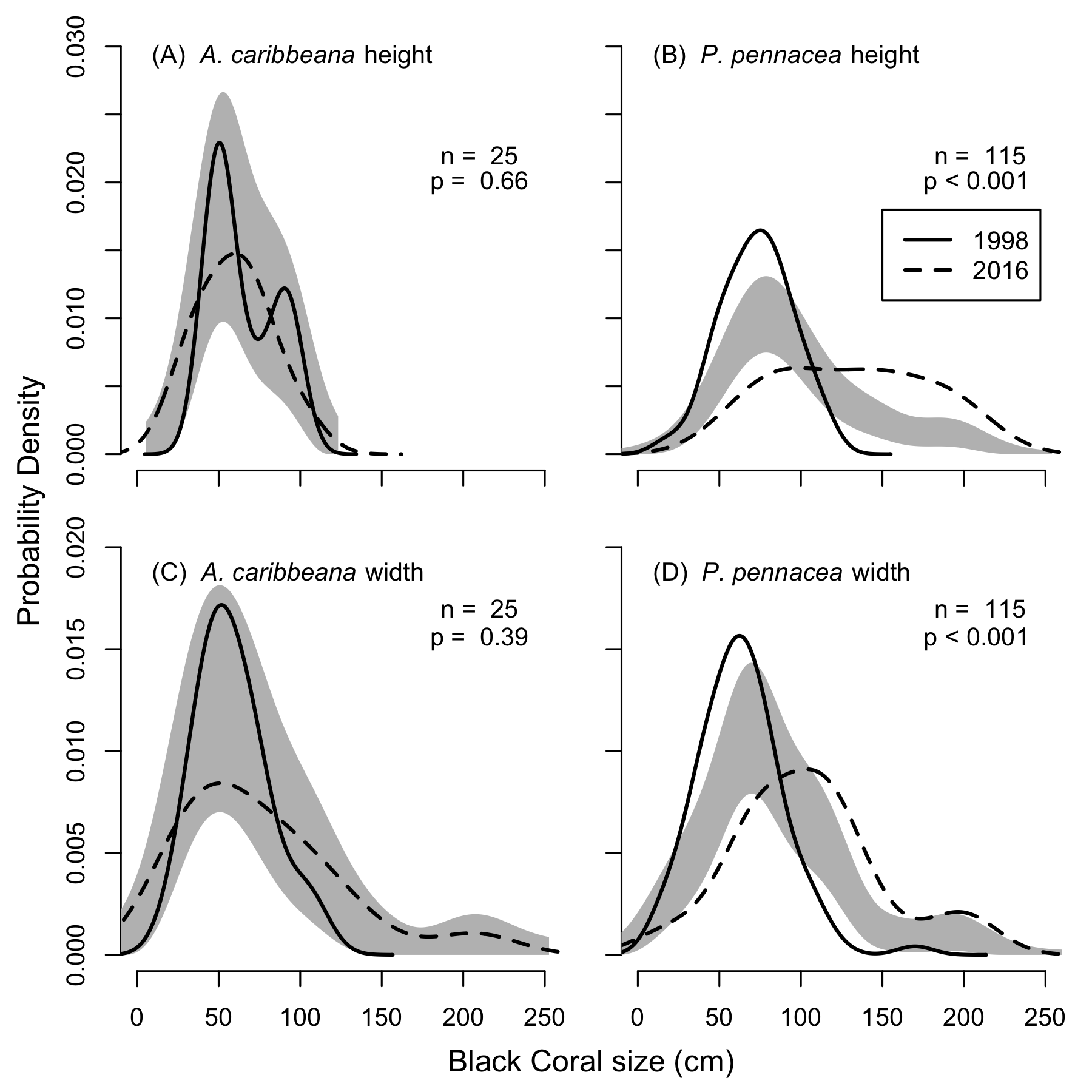

Supplement: Figure S2 — (A) A. caribbeana colony height, (B) P. pennacea colony height, (C) A. caribbeana colony width, and (D) P. pennacea colony width. Kernel density estimates were used, followed by a permutation test to identify differences between years. The grey shaded area indicates one standard error either side of the null model of no difference in colony size distribution based on year. Locations where the lines representing 1998 and 2016 are outside the grey zone indicate significant differences in the proportion of colonies of that size. n, number of colonies. [file peerj-06-5129-s002.png]

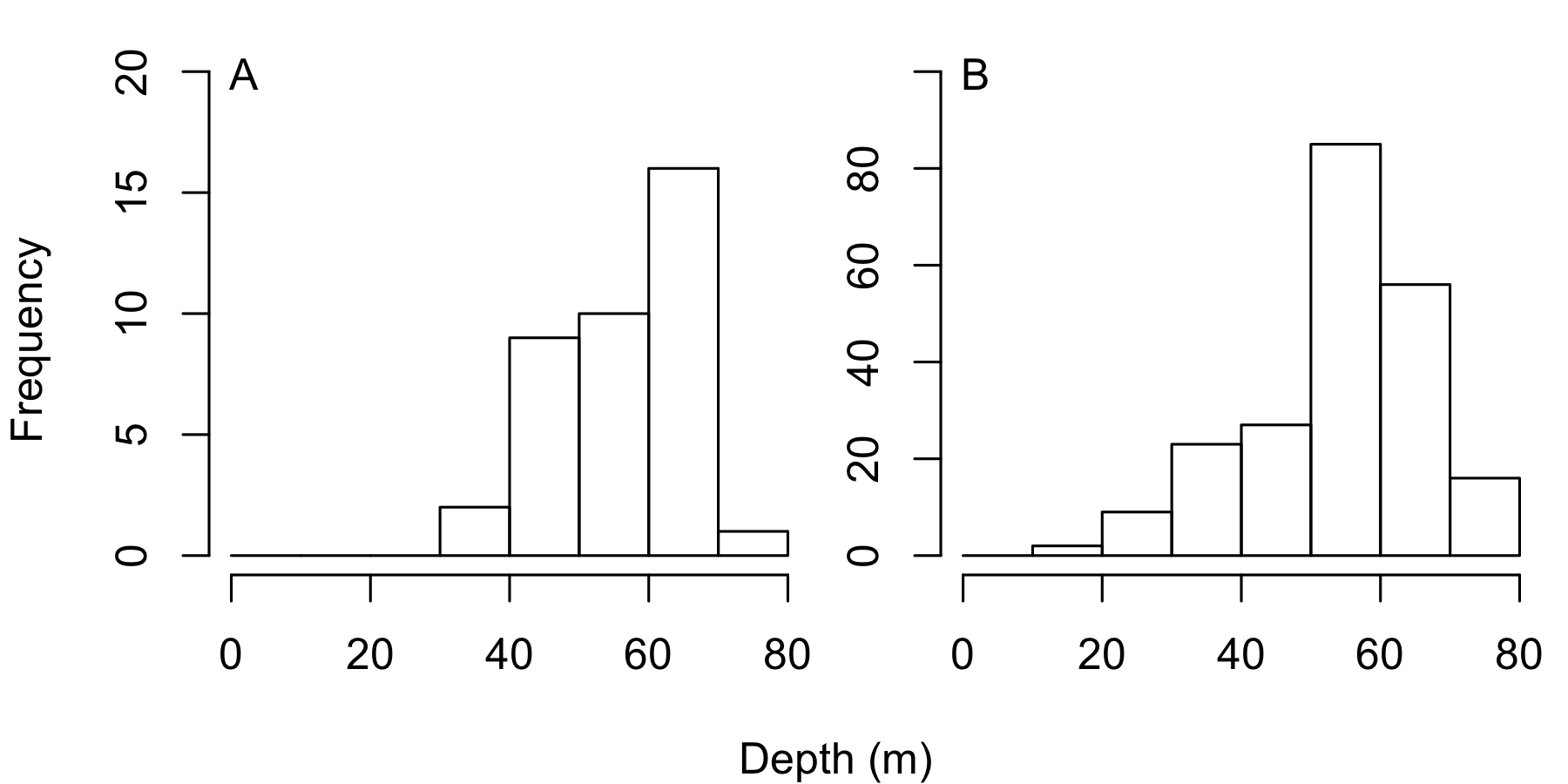

Supplement: Figure S3 — Survey effort was approximately equal at different depths in 1998 (Padilla Souza, 2000; Padilla Souza, 2004). [file peerj-06-5129-s003.png]
